# Colloidal 2D Lead Chalcogenide Nanocrystals: Synthetic Strategies, Optical Properties, and Applications

**DOI:** 10.3390/nano13111797

**Published:** 2023-06-03

**Authors:** Anton A. Babaev, Ivan D. Skurlov, Yulia A. Timkina, Anatoly V. Fedorov

**Affiliations:** PhysNano Department, ITMO University, Saint Petersburg 197101, Russia

**Keywords:** lead chalcogenide, nanocrystals, near-infrared spectroscopy, 2D nanocrystals, nanoplatelets, nanosheets

## Abstract

Lead chalcogenide nanocrystals (NCs) are an emerging class of photoactive materials that have become a versatile tool for fabricating new generation photonics devices operating in the near-IR spectral range. NCs are presented in a wide variety of forms and sizes, each of which has its own unique features. Here, we discuss colloidal lead chalcogenide NCs in which one dimension is much smaller than the others, i.e., two-dimensional (2D) NCs. The purpose of this review is to present a complete picture of today’s progress on such materials. The topic is quite complicated, as a variety of synthetic approaches result in NCs with different thicknesses and lateral sizes, which dramatically change the NCs photophysical properties. The recent advances highlighted in this review demonstrate lead chalcogenide 2D NCs as promising materials for breakthrough developments. We summarized and organized the known data, including theoretical works, to highlight the most important 2D NC features and give the basis for their interpretation.

## 1. Introduction

Colloidal lead chalcogenide nanocrystals (NCs) have become one of the most important materials for photonics applications in the near IR region of the spectrum due to their unique features, such as their tunable energy gap from red to mid IR, relatively high photoluminescence (PL), quantum yield (QY), highly efficient multiexciton generation (MEG), and solution processing [1,2]. Zero-dimensional NCs are usually referred to as quantum dots (QDs) and have been extensively applied in photovoltaics [3,4,5,6,7,8,9,10,11], thermoelectrics [12], and light-emitting diodes [13].

However, 0D systems were the first and relatively easy step in the global NC research and development process. For a few years, scientific attention was focused on two-dimensional systems due to their unique features inaccessible to their 0D counterparts. Two-dimensional NC thickness of a few atomic layers provides stronger 1D quantum confinement, which leads to narrow PL and absorption bands. Other unique characteristics of 2D NCs include high radiative recombination rates, giant oscillation strength [14], and huge surface/volume ratio. These features make them perfect building blocks for the new generation of light-emitting devices and lasers [15,16,17,18,19]. On the other hand, the theoretical modeling of 2D systems is complex and more complicated synthetic procedures are required to obtain a material with the desired properties.

Here, we want to summarize and analyze the progress on colloidal lead chalcogenide 2D NCs. Recent reports have demonstrated the superior optical and photoelectric properties of lead chalcogenide 2D NCs that outperform lead chalcogenide QDs in many fields. This brief review is organized into four parts. In the first part, we review the variety of synthetic approaches to 2D NC formation, with the main focus on the most common and effective protocols. The second part summarizes 2D PbX (X = S, Se, Te) NCs photophysical properties, starting with theoretical findings, then compares them with experimental results. The third part focuses on the applications of colloidal 2D PbX NCs. The fourth part summarizes the results of the review and gives an outlook for future studies.

**Figure 1:**
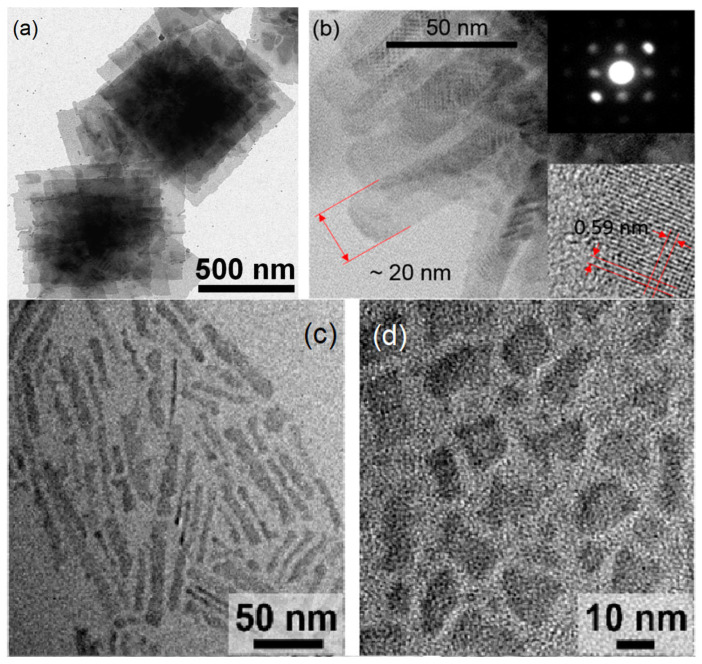
(**a**) TEM image of the stacked PbS NSs, reprinted with permission from [20]; (**b**) TEM images of PbS NRs, reprinted with permission from [21]. Copyright 2018 American Chemical Society; (**c**,**d**) TEM images of PbSe NPls, reprinted with permission from [22]. Copyright 2019 American Chemical Society.

In this review, we use different nomenclature for essentially very similar objects depending on the context. In theoretical work, 2D NCs are usually called quantum wells (QWs). In experimental work, 2D NCs with huge lateral sizes are called nanosheets (NSs) (Figure 1a), rectangular 2D NCs with a high aspect ratio are called nanoribbons (NRs) (Figure 1b), and 2D NCs with smaller lateral sizes are called nanoplatelets (NPls) (Figure 1c,d). Unless stated otherwise, in this review, the discussed 2D NCs are named according to the original papers and source naming therein.

## 2. Synthesis Strategies

The synthesis of 2D lead chalcogenide NCs is more complicated compared to the synthesis of their 0D counterparts, since a quasi-spherical morphology is the most common for rock salt crystal structures. The most common pathway to produce 2D materials is to use a precise combination of long-chain molecules to facilitate the crystal growth in a desired dimension. Other methods rely on the well-developed synthetic protocols for other 2D compounds and use the resulting NC as a template for lead incorporation. In this section, we discuss the most developed routines for colloidal 2D lead chalcogenide NC synthesis and summarize them in a chart.

**Figure 2:**
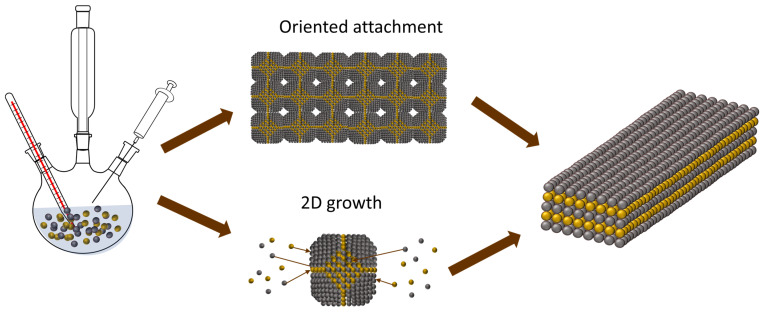
Schematized processes of oriented attachment and organometallic synthesis.

The first works devoted to the study of 2D lead chalcogenides considered epitaxial films [23,24]. A pioneering work on 2D colloidal lead chalcogenide NCs reported the synthesis of large PbS NSs by an oriented attachment of the small PbS NCs [20]. PbS NSs were obtained with a monodisperse 2.2 nm thickness and submicron lateral dimensions. In this reaction, conventionally synthesized quasi-spherical PbS QDs with 2.8 nm diameter were melted down by {110} facets in the presence of linear chloroalkanes. The resulting unstable “egg tray” structure rearranged to NSs, losing 21% of the initial QD thickness via atom migration. The concepts of oriented attachment and the organometallic synthesis are visualized in Figure 2. The main reason why the oriented attachment results in a 2D but not a 3D structure is still unclear. The authors of the original paper suggest the critical role of the oleic acid (OA) in forming ordered layers on the flat {100} planes, which drives the system to minimize the overall energy. This method is not exclusive to PbS 2D NCs and there are several papers reporting very similar 2D NC formation for PbSe NCs, where {110} facets and OA play key roles as well [25,26]. Further works optimized this method for field effect transistor (FET) and photodetector applications, discovering some unique phenomena that are discussed in the **Optical properties** section. Despite the good morphology and appropriate galena rock salt phase, the PL properties of such NSs were far from desirable.

Another reliable method to obtain large monodisperse ultrathin NSs is the single molecule precursor method. Akkerman et al. [27] used the decomposition of Pb-(thiocyanate)_2_ dissolved in OA, oleylamine (OLAm), and octadecene (ODE) to obtain 1.2 nm thick NPls. Khan et al. [28] used the Chugaev reaction of lead octadecyl xanthate and trioctylamine (TOA) to synthesize PbS NPls with sizes dependent on the reaction temperature. In both cases, the resulting 2D NC was characterized by an orthorhombic phase. This was further investigated by Macias-Pinilla et al. using a combination of optical spectroscopy, X-ray diffraction, selected area electron diffraction patterns, and DFT modeling [29]. It was found that orthorhombic PbS NPls possess an indirect bandgap. It is worth noting that such orthorhombic NPls still possess relatively weak and fast PL from a direct transition. Khan et al. [30] reported the rock salt structure of NPls synthesized using single molecule synthesis with lead hexadecyl xanthate and TOA at 60 °C. However, the PL properties of these NPls were not stated.

A vast number of synthetic procedures rely on organometallic synthesis, similar to QD synthesis. The resulting NPls usually have small lateral sizes (which are lower than the exciton Bohr radius) with a poor thickness distribution but a higher photoluminescence quantum yield (PLQY). Zhang et al. presented the first protocol for the direct synthesis of the PbS NPls in 2016 using PbO, bis(trimethylsilyl)sulfide, and OA [31]. The lateral size of the reported NPls varied from 200 × 20 nm to 200 × 100 nm, controlled by the temperature and OA amount, with thicknesses from 2.0 ± 0.3 nm to 3.6 ± 0.2 nm depending on the reaction time. The prolongation of the reaction time from 20 to 60 min increased the thickness from 2.0 ± 0.3 nm to 3.6 ± 0.2 nm, while higher temperatures and higher OA/Pb ratio lead to larger lateral sizes and more square-shaped NPls.

The PLQY of these NPls was about 6% compared to below 1% for oriented attachment NSs. Later, the best PLQY was reported by Antu et al. for PbS NRs synthesized using PbO, thioacetamide, OA, trioctylphosphine (TOP), and chloroalkane [21]. The NPls had 20 × 50 nm lateral dimensions and a thickness of about 2 nm. Their PLQY initially was 5.7%, which was improved to 34% after TOP passivation. Klepzig et al. synthesized PbSe NPls with a >7 nm lateral size and a PLQY of up to 37.4% [32]. The synthesis was performed using lead oleate and selenourea both at 0 °C and room temperature. The previous synthesis was optimized via passivation of the NPl surface with metal halides, which led to a PLQY of 64% [33]. It is worth nothing that the exact growth mechanism is not always well-studied and described in the papers; therefore, the exact synthesis type may be classified differently in various works.

Cation exchange (CE) methods have been widely exploited for 2D lead chalcogenide synthesis. Cd and Cu chalcogenide 2D NCs are typically applied as the template for CE. The conventional CE process exploits thermodynamic driving forces and kinetic aspects to extract cations from the chalcogen sublattice and replace them with desired cations. The most important parameters that are typically adjusted in most papers are the temperature, lead source, and molar ratio. Molecules that favor host cation extraction (TOP for Cu and OLAm for Cd) are selected on consideration of the Lewis acid and base principle. The exact optimal experimental parameters depend on the host NC properties that vary from synthesis to synthesis; however, some optimal parameters have been reproduced in several reports. PbBr_2_ was found to be an optimal Pb source for exchange from Cd-based NCs with a reaction temperature of 80 °C [22]. Despite the similarity of the Pb source type and temperature, the resulting NPls in work by Galle et al. [22] mainly maintained the host morphology, while in the synthesis by Zhou et al. [34], host NPls split into small particles. This could be explained by the different Pb source solvents and concentrations (0.065 mmol PbBr_2_ in 1 mL of OLAm compared to 4 mL of ODE [22] and 1 mmol PbBr_2_ in 5 mL of OLAm [34]), as well as the difference in the host NC properties. As expected, insufficient temperatures can result in partial exchange [35], while higher temperatures make the CE reaction go faster; however, overheating can result in NC degradation [36].

Besides the discussed synthetic strategies, a single paper on liquid phase exfoliation and photochemical synthesis has been published [37]. The published works are summarized in Table 1. In summary, there are a lot of developed methods to synthesize 2D lead chalcogenide NCs. The pros and cons of different approaches are not clear when looking at the synthesis alone. In the following sections on optical properties and applications, we will complete the picture to determine the progress in 2D lead chalcogenides and present an outlook for future studies.

## 3. Optical Properties

QWs or NSs are two-dimensional nanoobjects whose thickness is characterized by a nanometer scale and their lateral dimensions can be considered infinite. Their energy spectra are sets of two-dimensional sub-bands, in which the three-dimensional bands of bulk semiconductors are split due to the one-dimensional spatial confinement of the charge carriers. Unlike QWs, the lateral dimensions of NPls are relatively small and cannot be considered infinite, although they are still significantly larger than their thickness. Thus, the charge carrier motion in NPls is actually confined in all three dimensions and the energy spectra of the NPls electronic subsystem are discrete.

Bulk PbX at normal pressure exhibits a cubic face-centered lattice of the rock salt type [50]. The spatial symmetry group of these crystals is Fm3m, and the point symmetry group is $O_{h}$. Lead chalcogenides are direct-band multi-valley semiconductors with a fundamental optical transition located at the L-point of the Brillouin zone. Due to the spatial confinement in one dimension, the point group symmetry of PbX QWs is lower than in the bulk [51]. If their vertical interfaces are symmetric, then the point group symmetry of QWs is $D_{4h}$; if the vertical interfaces are asymmetric, then the point group is $C_{4v}$. Obviously, in the case of PbX NPls, their point group symmetry may be lower than $D_{4h}$ or $C_{c4v}$, since it will be determined by the shape of the NPls in the lateral plane. The exciton Bohr radius for PbS is 20 nm and for PbSe is 46 nm [52].

**Scheme 1:**
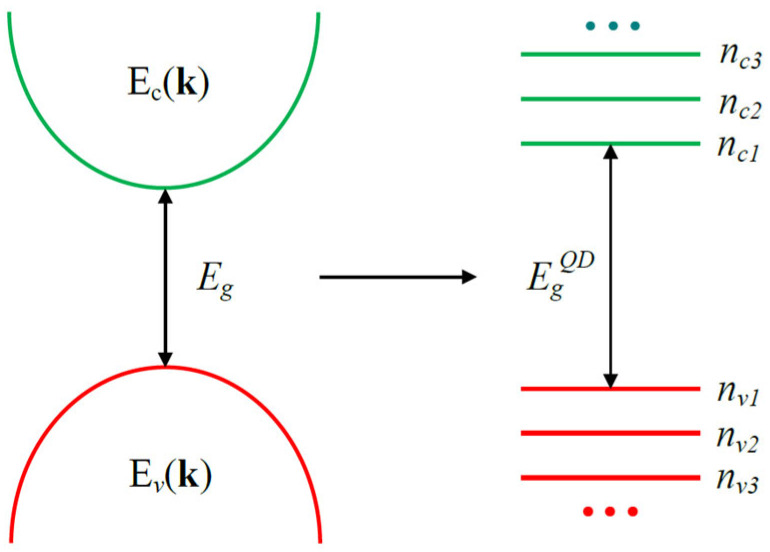
A qualitative scheme of the energy structure transformation of PbX nanomaterials due to the transition from one-dimensional to three-dimensional confinement. E_c_(**k**) and E_v_(**k**) are the continuous energy spectra of the conduction and valence bands of 2D PbX nanocrystals, where **k** is the two-dimensional wave vector, $E_{g}$ is the corresponding band gap, *n*_ci_ and *n*_vi_ (I = 1, 2, 3) are the quantum numbers of the size quantization discrete levels, and $E_{g}^{\mathrm{QD}}$ is the quantum dot band gap.

The band structure in 2D PbX nanocrystals is characterized by a continuous energy spectrum of the conduction *E_c_*(**k**) and valence *E_v_*(**k**) bands, where **k** is a two-dimensional wave vector lying in the lateral plane (e.g., Figure 3 in [53] or Figure 2 in [54]). In zero-dimensional PbX materials (quantum dots), the band structure represents discrete levels of size quantization (e.g., Figure 11 in [53]). Scheme 1 shows a qualitative transformation of a PbX nanomaterial’s energy structure due to the transition from one-dimensional to three-dimensional confinement.

Several approaches are used for the theoretical description of the electronic subsystem energy spectrum and the optical properties of quasi-2D semiconductor nanostructures of lead chalcogenides: the k·p perturbation theory effective mass method [55,56,57], the tight-binding method [53], and density functional theory (DFT) [29,37,46,51,54,58]. Each of them has its advantages and disadvantages. The most important results that can be compared with experimental data are discussed below.

Analytical expressions for the energy spectra and wavefunctions of the electronic subsystems of PbS and PbSe NSs were obtained by the effective mass method [57]. The calculations used a four-band model of a semiconductor (two conduction bands and two valence bands) in the spherical approximation, which corresponds to the L-point of the Brillouin zone. The energy of the fundamental transition, the effective masses, and the inter-band matrix element of the momentum were considered as fitting parameters. The calculations performed by the authors did not take into account the multipole nature of the energy spectrum and the Coulomb interactions of electrons and holes. In this regard, these results can only be considered as a preliminary basis for the development of a more accurate theory of the physical properties of PbX NSs.

The effective mass method was used to calculate the parameters of PbS and PbSe NSs at room temperature [55]. The calculations were performed using a four-band model (two conduction bands and two valence bands) with the band parameters of bulk materials. This model corresponds to the L-point of the Brillouin zone of bulk PbS and PbSe. The authors used the boundary condition that the envelopes of wavefunctions at the interfaces of NSs should be converted to null. To account for the Coulomb interaction in this work, the authors divided the motion of charge carriers into lateral and perpendicular motion at the NS interfaces. For the latter case, the Schrodinger equation was solved without the Coulomb interaction, which was afterwards included as a perturbation. An effective two-dimensional Coulomb potential was proposed, which was used to describe the lateral motion of charge carriers and calculate corrections to the solutions of the Schrodinger equation for motion perpendicular to the interfaces. The authors state that their calculations agree well with the experimental data; however, they used data for NSs with a thickness of >2 nm and ignored some published experimental results, such as Schliehe et al.’s work [20].

Among the approaches used to describe the physical properties of lead chalcogenide NSs and NPls, calculations performed by the tight binding method should be noted, for example, the work by Allan et al. [53]. In this paper, the energy spectra of the electronic subsystem and the optical properties of PbSe quantum wells with a (001) surface were obtained taking into account their multi-valley band structure. The authors state that the energy of the fundamental optical transition or bandgap (*E_g_*) is inversely proportional to the quantum well thickness (*Z*). This behavior can be explained by the almost linear dispersion of the bulk bands energy near the Brillouin zone L-point. It is important to note that the dependence of *E_g_*(*Z*) for quantum wells with an even and odd number of layers is different. In [53], the formula for *E_g_*(*Z*) is

(1) $$E_{g}\left(Z\right)=E_{g}\left(\infty \right)+\frac{1}{1.2125\cdot Z+0.3326},$$

where Z is the thickness of the quantum well in nanometers and *E_g_*(∞) is the bulk bandgap. This formula gives the average value of *E_g_* (in eV) for the number of even and odd layers. In addition, it was shown that the imaginary part of the frequency-dependent permittivity (absorption of electromagnetic radiation) has a strong anisotropy. The disadvantages of Allan’s work are that the calculations did not take into account the Coulomb interactions between electrons and holes, which is extremely important for ultrathin quantum wells [59,60,61], and the possible dependence of the tight binding parameters on the thickness of PbSe quantum wells.

Another interesting approach often used to describe the energy spectrum of the electronic subsystem and the optical properties of PbX NSs is ab initio calculations based on DFT [29,37,46,51,54,58]. The Quantum Espresso software package (the current 7.2 version [62]) was used to perform DFT calculations of the electron density of states and, consequently, the fundamental optical transition energy *E_g_* in PbS NSs as a function of their thickness Z [46]. Perdew–Burke–Ernzerhof (PBE) norm-preserving pseudopotentials were used in the calculations for Pb and S [63]. Calculations were performed for the supercell model in slab geometry. The authors claim that the obtained results are very close to the experimentally obtained dependence:

(2) $$E_{g}\left(Z\right)=E_{g}\left(\infty \right)+\frac{1}{0.99\cdot Z+1.18},$$

where $E_{g}$ is given in eV and *Z* in nm. However, this statement should be treated with great caution, since, as the authors note, they failed to obtain reasonable absolute values of *E_g_*(*Z*). They were able to identify only the trend in changes in *E_g_*(*Z*) depending on *Z* due to the well-known bandgap problem inherent in DFT calculations [64]. In addition, the calculations did not take into account *E_g_*(*Z*)’s dependence on temperature. Without such considerations, DFT calculations will only describe the object at zero temperature. Again, the theoretical description developed by the authors does not take into account the Coulomb interactions between electrons and holes. Finally, it should be noted that the dependence of *E_g_*(*Z*), although inversely proportional to Z, differs markedly from the dependence obtained by Allan et al. in Equation (1) [53]. In particular, no difference was found between *E_g_*(*Z*) for NSs with even and odd numbers of layers. We would like also to point out that nothing is said about taking into account relativistic effects in this paper.

In Wan et al.’s [54] work, DFT calculations were performed based on the projector augmented plane waves method (PAW) [14] using the Vienna Ab initio Simulation Package (VASP) [65]. PbS NS band structure calculations were performed using the hybrid functional of Heyd–Scuseria–Ernzerhof (HSE) [66] and PBE, taking into account spin–orbit coupling (SOC). A comparison of the obtained results with the experimental data [67] showed that the use of HSE+SOC gives values of the fundamental optical transition closer to the experimental values than the use of PBE+SOC. It was shown that the fundamental transition energy $E_{g}\left(Z\right)$ oscillates depending on the number of layers in PbS NSs, i.e., the dependence on the thickness of the NSs, Z, for an even and odd number of layers is different, which is consistent with Allan et al.’s results [53]. When calculating the band structure of the PbS monolayer, it turned out that the fundamental transition energy is 0.24 eV, which is unexpectedly low. The authors explain this fact by the presence of a strong crystal field [68] and the influence of pressure [69]. In addition, the effect of uniaxial and biaxial deformations on the energy structure of PbS NSs was analyzed. Although the absolute values of $E_{g}\left(Z\right)$ obtained in [54] are quite close to the experimental data, they should be treated with caution due to the bandgap problem. Among the drawbacks of the work by Wan et al., it should be noted that the theoretical description does not take into account the Coulomb interactions between electrons and holes as well as temperature effects.

**Figure 3:**
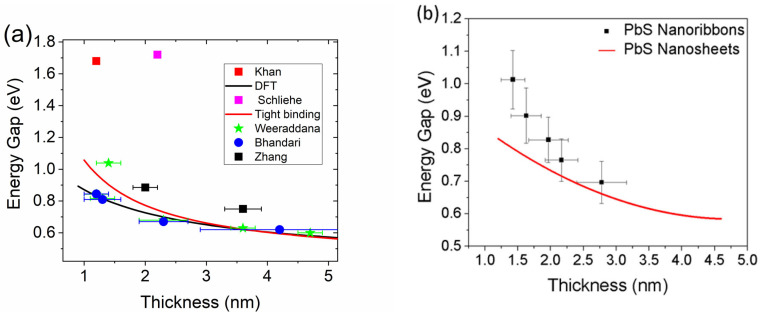
(**a**) Experimental data from [20,27,31,70] plotted together with the curves of Equations (1) and (2); (**b**) experimental data plotted with the curve of Equation (2), reprinted with permission from [21]. Copyright 2018 American Chemical Society.

To compare the experimental data with theoretical data, we present Figure 3a, where the known data on NSs [20,27,31,70] are compared with predictions from Equations (1) and (2). As can be seen, the theory poorly fits the experimental results, which indicates the importance of the unaccounted parameters discussed above. Namely, Khan et al.’s [28] NS deviation from theoretical data could be caused by the orthorhombic PbS structure observed for this NS. On the one hand, the orthorhombic structure has an indirect bandgap [29], which should not yield any PL signal. On the other hand, the specific synthesis conditions for the same procedure yield rock-salt-structured NSs. However, in works by Zhang et al., Schliehe et al., and Weeraddana et al., NSs had a rock salt structure and their bandgaps also poorly fit with the theory. Thus, discrepancies between the theory and experiment cannot be explained in terms of the crystal phase alone. Interestingly, three of four NSs from Weeraddana et al.’s work perfectly match the DFT results [46], where a 0.1 nm change in the thickness dramatically shifts the band gap energy. Another important thing to note is that these NSs have a 20 × 20 nm lateral size, which seems to have a negligible impact on the NS energy structure despite the fact that weak confinement should appear for NCs of that size. It is also in striking contrast with Antu et al.’s work (Figure 3b) [21], where NRs with lateral sizes of 50 × 20 nm showed a much higher bandgap energy.

The published NC PL properties are summarized in Table 2. Most researchers report an inhomogeneous broadening of the PL spectra due to the dispersion in thickness and/or lateral sizes. The other possible reason is surface-trap-assisted PL, which is well known for lead chalcogenide QDs [71]. The PL decay features (namely the excitation-intensity-dependent PL decay and PL spectra) indicate the same processes as PbS NSs, as noted by Zhang et al. [31]. Skurlov et al. [72] identified two radiative states in PbSe NPls, one of which was ascribed to trap-related PL. In general, surface trap states are one of the main NC non-radiative energy loss pathways. The difference in the PL lifetime among the published papers is mainly caused by different surface passivation, while higher PLQYs correlate with higher PL lifetimes, as it should be according to the well-known relation between PLQYs and radiative recombination times [73]. In 2D NCs, the surface/volume ratio is usually high, which leads to a low PLQY. Numerous surface post-treatment strategies have been developed for optical property improvement, namely CdCl_2_ treatment [32,33,45], TOP treatment [21], CdS shell growth [31], and lead halide salt treatment [33].

Defects can originate from various sources: non-passivated non-stoichiometric facets or intrinsic defects typically induced by external molecules [74]. There are usually two pathways to mitigate them: inorganic shell growth and careful control of the surface ligands. For the latter, the appropriate surface ligand treatment is determined by NC facets. The DFT calculations for PbS QDs show that {100} facets are stochiometric and thus self-passivated, while the {111} facet is Pb-rich and can be completely passivated with oleate and hydroxide anions [75]. Lead atoms in the {110} facets typically present on PbS 2D NC edges (see the synthesis section) are well passivated with OA, while sulfur atoms are not, which leads to hole trap formation. Sulfur atoms in turn can be passivated using TOP. Metal halide passivation is more complicated though; depending on the treatment parameters, the results can differ. For example, Galle et al. [22] used XPS analyses to detect the passivation of the PbSe NPL surface with an additional PbBr_2_ layer that presumably formed via Z-type addition. The use of metal halide treatment for PbSe revealed that passivating with both Z-type and X-type ligands (halide anions) is required for PLQY improvement [33].

**Table 2:** PL properties of known lead chalcogenide 2D NCs and QDs.

Original Name	Ref.	PL Peak Position (eV)	FWHM (meV)	PLQY (%)	PL Lifetime (ns)	Thickness	Lateral Sizes (nm)	Crystal Structure
PbSe NPls	[32]	0.85–1.25	100–200	37	1200	0.8 ± 0.1 nm	3–6.4	
PbSe NPls	[22]	0.8–0.93	80–240	15	700–2700	3–6 ML	∼10–30	rock salt
PbSe NPls	[40]	1.02	207			4 ML	∼15 × 50	rock salt
PbS NRs	[21]	0.69–1.13	130–180	34	96–372	1.4–2.8 nm	20 × 50	rock salt
PbS NSs	[42]	3	113			<5 nm	>1000	zinc blend
PbS NPls	[28]	1.66–1.69	106–156		59 to 10	1.8–2.3 nm	3.5–21	ortho-rhombic
PbSe NPls	[72]	0.95	108		600	5 ML	28 × 15	
PbS NPls	[76]	1.69	~122			1.8–2.3 nm	47.1 ± 6.5	ortho-rhombic
PbS NPls	[45]	1.73	142	1.4/19.4		1–2 nm	7 × 10	rock salt
PbS NSs	[46]	0.62–0.84				1.2–4.6 nm	>1000	rock salt
PbSe NPls	[25]	∼1.13	∼180	∼0.1		∼2 nm	∼30	rock salt
PbSe NPls	[33]	1.39		18–64	∼2000	0.8 ± 0.1 nm	2.7 × 4.9	rock salt
PbS/CdS Core/Shell NSs	[67]	0.75, 0.85 after CE	∼120			3.3 ± 0.1 nm, 2.1 nm after CE	>100	
PbS NSs	[27]	∼1.08	∼120			1.2 ± 0.3 nm	∼180 × 35	rock-salt
PbS NSs	[70]	0.6 to 1	∼70			4.7 to 1.2 nm	∼20 × 20	
PbSe_1−x_S_x_ NPls	[34]	from 0.9 to 1.05	>200	60	1500	∼2 nm	4–6	rock salt
PbS NSs	[49]	0.62–0.73				8–12 ML	100 × 100	
PbS and PbS/CdS NSs	[31]	0.75 to 0.86		6/11	40/190	2.0 ± 0.3 to 3.6 ± 0.2 nm	200 × 20/200 × 50/200 × 100	
**QDs**	**Ref.**	**PL Peak position (eV)**	**FWHM (meV)**	**PLQY (%)**	**Decay Time (ns)**	**Size (nm)**		**Crystal Structure**
PbSe QD single	[77]	1.55	100	30	n/a	~3		Rock salt
PbS QD ensemble	[78]	0.65–1.3		5-50	n/a	n/a		Rock salt
PbS QD ensemble	[79]	0.91	105		n/a	2.6		Rock salt

A comparison of PbX 2D NC and PbX QD photoluminescent properties reveals some prospects. Reported 2D NC FWHM values vary from paper to paper in the range between 70 meV and 240 meV. This is in stark contrast with CdX 2D nanocrystals, e.g., CdX NPls can exhibit PL as narrow as 41 meV for CdSe [80]. Skurlov et al. [72] touched upon this phenomenon in their research and found that the difference lies mostly in the inhomogeneous broadening. They suggested three possible reasons: quantum confinement in the lateral dimensions of the NPls, lattice distortion during cation exchange, and the influence of the surface trap states. However, it is worth noting that for single PbS QDs, the PL FWHM was ~100 meV (which was even higher for NPls in solution) [77], while for PbS NSs in colloidal solution, the FWHM was ~70 meV [70], which means that potentially it is possible to produce PbX 2D NCs as NIR emitters with a narrow band.

MEG has been observed for PbS NSs [81]. MEG or carrier multiplication phenomena occur when one absorbed photon can generate more than one electron–hole (e–h) pair. When NCs absorb a photon with an energy higher than their double band gap, the excess energy is converted to kinetic energy that can dissipate by electron–phonon scattering or excite another e–h pair. The second case is highly desirable for photovoltaic applications since it is a way to overcome the Shockley–Queisser limit [82] which is exciting for PL applications as the PLQY can exceed 100% in this case. Aerts et al. [81] studied 4, 5.9, and 7 nm thick PbS NSs (with band gap energies of 0.83, 0.64, and 0.56 eV, respectively) with pump–probe spectroscopy and found the MEG threshold to be 3 eV for all NS sizes and found an MEG efficiency comparable with bulk PbS. While this result is inspiring, the reasons why the MEG threshold is this high are unclear and further investigation is required.

Usually, the optical and electrical properties of a semiconductor are dependent on the temperature. In the case of NCs with a high surface-to-volume ratio and henceforth an abundance of surface defects, the role of the temperature increases. Temperature-dependent studies are a versatile tool for characterizing NCs, including 2D NCs. Cooling down a single-nanosheet-based FET below 200K led to a drastic increase in the FETs On/Off ratio by five orders of magnitude [83]. Temperature-dependent measurements can also reveal additional information about PL, such as the nature of PL line broadening. It was found that in cation-exchanged PbSe NPls, a wide PL band arises from the large inhomogeneous broadening associated with internal emission mechanisms [72]. The PL intensity of NPls is amplified during cooling. Zhou et al. [34] reported an eight-fold increase in the PL intensity after cooling ultrasmall PbSe_1-x_S_x_ NPls from 300 to 100 K, which was associated with the suppression of charge carrier trapping by defects/traps and phonon-assisted thermal escape. In both papers, the NPls PL redshifts and the FWHM decreases during cooling, which is in good agreement with findings for PbX QDs [84].

## 4. Applications

**Figure 4:**
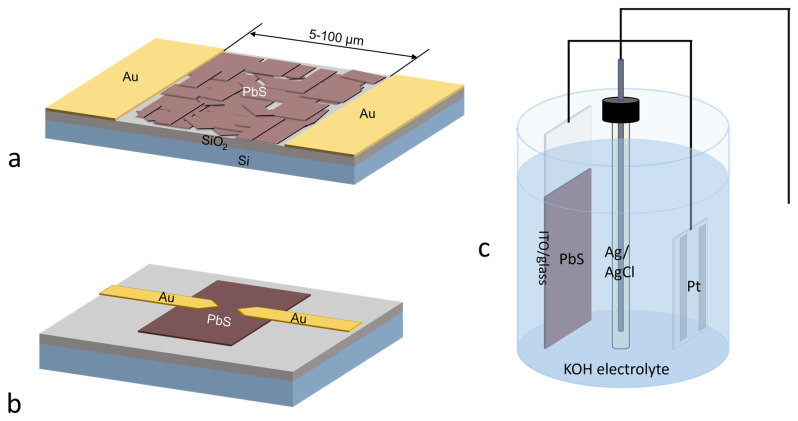
Scheme of a typical FET/photoresistor structure (**a**); an FET on a single NS (**b**); a photoelectrochemical photodetector (**c**).

Even in early works, the electric properties of 2D lead chalcogenide NCs have been of great interest. In most cases, the structures applied in electric and/or photoelectric studies of 2D NCs are similar to those depicted in Figure 4. In all the works mentioned below, gold is used for contacts and the gap between contacts is 5–100 µm, unless stated otherwise.

The first synthesized PbS NSs [20] demonstrated a two orders of magnitude conductance improvement under 2.0 mW/cm^2^ 532 nm laser illumination. However, the observed effect was not only due to e–h pair photogeneration, but also due to surface trap filling as well. Bielewicz and Dogan et al. synthesized several-micron-wide NSs using the oriented attachment method and used these individual sheets as an FET [44,83]. All devices showed p-type behavior as a consequence of the ohmic contact for holes and Schottky barriers for electrons at the metal–NS interface. The NS shows remarkable photoelectric properties, with 0.417 cm^2^V^−1^s^−1^ charge mobility, 72.06 mS/cm conductance, and a 7404-fold current increase under 1 mW/cm^2^ illumination at 77 K. Upon varying the sheet thickness, they found that thinner NSs show a steep sub-threshold swing and a higher On/Off current ratio. It is worth noting that the optical measurement of the charge mobility in PbS NSs results in values of 550–1000 cm^2^V^−1^s^−1^ depending on the NS thickness [85]. The dramatic difference in the optical and electrical measured values could be the result of the abovementioned metal–semiconductor interface’s influence on overall NS properties. Small colloidal NPls obtained through CE from CuS NPls were applied to build an FET by Sonntag et al. [38]. The ideal case for such an approach is an active layer, with the majority of PbS NPls lying face down. As the NPls are capped with long-chain organic molecules, proper surface passivation via ligand exchange is one of the most important aspects for good conductance. To achieve this, the authors applied LiI or PbI_2_ ligand exchange with annealing at 200 °C and 350 °C for FET fabrication. However, the NS film resistance remained relatively high (2.7 × 10^5^ Ω sq^−1^ at best). The FETs show p-type conductance with a ∼60 On/Off current ratio and a threshold voltage of −3.8 V. The linear and the saturation charge mobilities were 0.02 cm^2^V^−1^s^−1^ and 0.012 cm^2^V^−1^s^−1^, respectively.

Orthorhombic PbS NSs synthesized by Akkerman et al. [27] were tested as active layers in photoconductors. A 400 nm thick film was prepared using 1-ethyl-3-methylimidazolium iodide for ligand exchange. The resulting device shows a responsivity of up to 0.1 A W^−1^ and a detectivity of up to 1.3 × 10^9^ Jones with good bending and humidity resistance on a flexible substrate. Wu et al. [36] applied Cu-doped PbS NPls for photodetector fabrication. A ~57 nm film was deposited using 3-mercaptopropionic acid treatment and annealing at ~250 °C for 1 h in an N_2_ atmosphere. The resulting device shows a ~1739 AW^−1^ responsivity at 808 nm irradiation, a specific detectivity of ~ 2.55 × 10^11^ Jones, and a resistivity of 8.04 Ohm·cm. The authors explain such a high performance by the unique NPl facet distribution ({111} basal planes), higher NPl packing densities, and the ligand choice. Galle et al. [40] used PbSe NPls obtained by simultaneous CE and ligand exchange from CdSe NPls for detector fabrication. A 100 nm thick layer of I^−^-capped NPls was spray-coated onto an interdigitated Pt-patterned glass substrate for device fabrication. The device yields responsivities of 0.180 AW^−1^ at 450 nm irradiation and 0.017 AW^−1^ at 1000 nm irradiation. The device shows a fast photoresponse (*f_3dB_*∼10 kHz) that outperforms colloidal PbSe QD-based analogs and is comparable to other PbSe NC-based devices. The ultrafast photoresponse was observed with more precision by Gao et al. [37] for PbS NPls obtained by liquid-phase exfoliation. Transient pump–probe spectroscopy yielded exciton lifetimes of 2.6 ± 0.1 and 6.2 ± 0.8 ps at 500 and 740 nm excitation, respectively. Furthermore, they fabricated a photo-electrochemical photodetector using the PbS NPls as a working electrode and applied DFT to better understand the underlying processes. The photo-electrochemical photodetector shows a photo-responsivity of 27.81 mAW^−1^, a detectivity of 3.96 ×10^10^ Jones, and excellent long-term cycling stability.

To summarize the photoelectric applications, it is valuable to benchmark the 2D PbX NC devices against PbS QD-based analogs. The same simple structure shown in Figure 4 but with a PbS QD active layer yields a detectivity of 1.8 × 10^13^ Jones and a responsivity of >10^3^ A W^−1^ with a photoresponse of 20 Hz [86]. Reported photoresponse times are slower than those reported for NPls (∼10 kHz) [37] used in the same simple device. The device response time is crucial and is one of the main factors in determining the usability of a device. It is important to note that improving the response time negatively impacts the responsivity, which limits further development. To overcome this limitation for QD-based devices, researchers employed heterojunction-based devices to achieve an adequate response time without impairing the responsivity. After about two decades of progress, a PbS QD/double-wall carbon nanotube photoconductor device achieved a responsivity of 75 AW^−1^ with a response time of 200 µs under low power 433 nm laser irradiation. To date, there have been no successful attempts to engineer such a complicated structure for 2D PbX NCs. However, the mere fact that pure 2D NCs can yield a fast response is intriguing in itself.

We should also mention the work by Zhang et al. [58], in which the authors investigated the effects of SO_2_ and Cl_2_ adsorption on the electronic structure of single- and multi-layer PbSe NSs based on DFT calculations using the VASP software package (the current 6.4.1 version) and the PBE functional. It turned out that the electronic structures of PbSe NSs adsorbed with SO_2_ or Cl_2_ differ from each other, which makes PbSe NSs a promising material for sensors. Other lead chalcogenide applications also deserve to be mentioned. PbS NPls were used for improving perovskite crystallization in solar cells [76]. PbSe NSs were utilized for harmonic mode locking in fiber lasers [43]. Li-ion battery performances were also enhanced using PbSe/PbS NSs [48].

Some research groups have tuned the PL of NIR-emitting PbX NCs (including 2D NCs) to overlap with biological tissue transparency windows for further applications [34]. This, however, raises the question of NC toxicity. For PbX compounds, the most concerning element is Pb, which is associated with neurotoxicity, hepatotoxicity, and nephrotoxicity [87,88]. The European Union restricts the lead content in electronics to 0.1% wt [89]. Considering 2D PbX nanocrystals, there are no research data on their toxicity. However, as the chemical composition is the same for more widely studied PbX quantum dots, some parallels might be drawn. A universal strategy to reduce QD toxicity for in vivo and in vitro applications is the growth of an inert shell, which reduces the detrimental effects [90,91]. Reducing the use of toxic reagents in NC post-synthetic processing should also be considered, as it reduces the amount of toxic waste [92]. An extensive discussion on NC toxicity goes beyond the scope of this review, and we would recommend studies on the topic such as the work of Yong et al. [93].

## 5. Conclusions and Outlook

Overall progress on lead chalcogenide 2D NCs is not as explosive as for perovskite- or Cd-based materials, but recent advances give solid reasons to dedicate more efforts to their study. Two-dimensional NCs have promising optical features, such as higher radiative recombination rates and potentially lower FWHMs, when compared to 0D NCs. This can lead to the development of near-infrared emitting light sources with a narrow spectral linewidth.

The presented papers show that the operating properties of 2D NC-based FETs and photodetectors can overcome their QD-based counterparts, allowing their employment in flexible devices [27]. The high carrier mobility shown in theoretical and experimental papers is a key parameter for fast devices with a high bandwidth [94]. At the moment, the presented devices are but starting concepts that can only provide insight into the material’s potential. Extensive studies on QD-based devices can be used as a background for further research. It is exciting to see what can be achieved for 2D NC-based devices with the extensive use of heterojunctions and optimized device architecture, as well as the implementation of carbon materials for heteronanostructure fabrication.

There is a lack of theoretical works on lead chalcogenide 2D NCs. New efforts should focus on the improvement of modeling, and we propose several directions. Tight binding calculations can be improved by taking into account the Coulomb interactions between electrons and holes as well as the dependence of the tight binding parameters on the PbX quantum well thickness. The improved DFT modeling of PbX QWs and NSs physical properties should involve the use of the most accurate and new pseudopotentials as well as the implementation of advanced calculation methods similar to those described in the work of Kirchner-Hall et al. [64]. This should alleviate the bandgap problem. In addition, it is necessary to develop methods that take into account the temperature effects and the Coulomb interactions between electrons and holes. An important direction in modeling the energy structure and optical properties of PbX QWs and NSs is the use of many-body perturbation theory [95] and dynamic mean field theory [96]. As for calculations based on k·p perturbation theory, it is necessary to develop an approach that will simultaneously take into account the multi-valley energy structure, the spin–orbit interactions, the Coulomb interactions between electrons and holes, and the dependence of the theory parameters on the sizes and shapes of PbX QWs, NSs, and NPls. This is especially important for NPls, which consist of a finite but very large number of atoms (several thousand). Obviously, an adequate quantitative description of lead chalcogenide 2D NCs physical properties is possible only if the results obtained by different theoretical methods can be compared with each other as well with the experimental data.

Another issue is the PLQY of 2D PbX nanocrystals. In some cases, it can reach above 60%; however, usually it is either rather low or not reported. In order to employ such NCs, it is necessary to improve their PLQY. The pathways to achieve this are synthesis optimization and efficient surface trap passivation. Development of new theoretical models and simulations of NPl structures could be helpful for surface passivation strategies to evolve from empirical exhaustive searches to predictable functionalization.

There are no systematic studies of the ligand’s influence on lead chalcogenide 2D NC energy structures, which is essential for application in existing solution-processed devices. One more important subject that still has not been properly investigated is single lead chalcogenide 2D NC spectroscopy. Such a study will be valuable for a deeper understanding of the photoexcitation dynamics of 2D NCs, including blinking processes, and for the development of single quantum emitters based on lead chalcogenide 2D NCs.

Finally, the most obscure theme is PbTe 2D NCs. There are only a few papers where such materials are studied [97,98,99], but they mostly lack proper optical characterizations, and it is not possible to be confident on the relevance of the reported results for the reviewed topic. Proper PbTe 2D NC synthesis and characterization will be helpful for lead chalcogenide 2D NC studies.

## Figures and Tables

**Table 1:** The published original synthesis procedures.

Original Name	Ref.	Synthesis Method	Thickness	Lateral Sizes, nm	Crystal Structure
PbS NPls	[38]	CE (PbO, OA, 100 °C) from CuS	5 nm	40	rock salt
PbSe NPls	[32]	Direct (Pb(OA)_2_, HOA, 0 °C)	0.8 ± 0.1 nm	3–6.4	
PbS NSs	[39]	Direct (photochemical)	1.4 nm	>200	
PbSe NPls	[22]	CE (PbB_r2_, OlAm, 80 °C) from CdSe	3–6 ML	∼10–30	rock salt
PbSe NPls	[40]	CE + ligand exchange (PbI_2_ and NH_4_I) from CdSe	4 ML	∼15 × 50	rock salt
PbS NRs	[21]	Direct (PbO, OA, thioacetamide, DMF 100 °C)	1.4–2.8 nm	20 × 50	rock salt
Biconcave PbS NPls	[41]	CE from Cu_1.94_S	∼2.5 nm	∼25	rock salt
PbS NSs	[42]	Direct (Pb(OAc)_2_, thiourea, sodium dodecyl sulfate, NaOH, 60 °C)	<5 nm	>1000	zinc blend
PbS NPls	[28]	Single molecule precursor (Chugaev reaction with lead octadecyl xanthate, TOA, 80–90 °C)	1.8–2.3 nm	3.5–21	orthorhombic
PbSe NSs	[43]	Direct (PbCl_2_, selenium powder, 220 °C)		>100	
PbS NSs	[44]	Direct (thioacetamide, DMF, TOP, PbOAc, OA)	4, 6, 16, 23 nm	>100	rock salt
PbS NPls	[45]	Direct (lead oleate, octylamine, thiourea, OA, room temperature)	1–2 nm	7 × 10	rock salt
PbS NPls	[37]	Liquid-phase exfoliation	3.7 ± 0.9 nm	11.2 ± 1.7	
PbS NPls	[36]	CE (lead oleate) from Cu_2_S	~3.5 nm	~120–150	
PbS NSs	[20]	Oriented attachment	2.2 nm	>200	rock salt
PbS NSs	[46]	Oriented attachment	1.2–4.6 nm	>1000	rock salt
PbSe NPls	[25]	Oriented attachment (PbCl_2_ and Se powder react in a mixture of alkyl amines)	∼2 nm		rock salt
PbS NSs	[27]	Single molecule precursor (Pb(SCN)_2_, ODE, OA, 165 °C)	1.2 ± 0.3 nm	∼35–180	orthorhombic/rock salt
PbSe_1−x_S_x_ NPls	[34]	CE (var. PbCl_2_, PbBr_2_, PbI_2_, Pb(oleate)_2_) from CdSe_1−x_S_x_	∼2 nm	4–6	rock salt
PbS NSs	[47]	Coalescence of PbS nanowires	1.8 nm	200-20000	
PbSe NC	[26]	Direct (PbO, OA, Se, TOP, 130 °C)	4.3 ± 0.5 nm	200	
PbSe/PbS NSs	[48]	Direct (one pot autoclave)	10–30 nm	250–350	
PbS NSs	[49]	Oriented attachment (PbO modification)	8–12 ML	100 × 100	
PbS and PbS/CdS NSs	[31]	Direct ((PbO) and bis(trimethylsilyl)sulfide, 90 °C)	2.0 ± 0.3/3.6 ± 0.2 nm	200 × 20, 200 × 50, 200 × 100	
2D PbS NC	[30]	Single molecule (lead hexadecyl xanthate and TOA at 60 °C)	∼2 nm	180 × 40	rocksalt

## Data Availability

No new data were created or analyzed in this study. Data sharing is not applicable to this article.
